# Intussusception in a child with Acute Lymphoblastic Leukemia: a remarkable presentation with literature review – a case report

**DOI:** 10.1186/s43046-021-00079-z

**Published:** 2021-07-29

**Authors:** Ankur Majumder, Sunayana Misra, Vijay Kumar, Shilpa Khanna Arora

**Affiliations:** 1 Department of Pathology, ABVIMS & Dr. RML Hospital, 110001 New Delhi, India; 2 Department of Pediatrics, ABVIMS & Dr. RML Hospital, New Delhi, India

**Keywords:** Intussusception, Acute Lymphoblastic Leukemia, Gastrointestinal Complications, Hematopathology, Surgery

## Abstract

**Background:**

Gastrointestinal complications are not uncommon in patients of Acute Leukemia. Intussusception as a complication in leukemia, although described, is exceedingly rare. Also, it is usually seen after chemotherapy and not as a part of the native disease process. This case report aims to highlight such a rare association which warrants clinical and pathological attention.

**Case presentation:**

A 14 year old male presented with an acute abdomen. Initial routine investigations revealed a deranged blood picture. On further examination of bone marrow aspirate, biopsy and detailed immunohistochemical studies a diagnosis of B-Acute Lymphoblastic Leukemia (B-ALL) was made. Concurrent ultrasound of the abdomen to find a cause for severe abdominal pain revealed an Ileo-colic intussusception. The patient was started on steroids; however he succumbed to his illness after two days, before surgery could be attempted.

**Conclusion:**

Rare presentations of relatively common diseases are a hurdle for timely and effective medical intervention. Although a rare condition in itself in leukemic patients, the occurrence of Intussusception in this particular patient, especially when no chemotherapy was initiated, is a very rare event. This case report was made to add to the relatively scarce literature available on this particular association. As it is a surgically treatable condition and since delay in diagnosis may lead to poorer prognosis, possibility of co-existence of ALL and intussusception should be borne in mind by all treating physicians and hematopathologists for effective patient care.

## Background

Acute lymphoblastic leukemia (ALL) is a malignant transformation and proliferation of lymphoid progenitor cells in the bone marrow, blood and extramedullary sites. Eighty percent of ALL occurs in children [1]. Intestinal complications in ALL have been documented in literature, although in the majority of cases they are clinically silent [2]. Previous studies have noted direct infiltration of the intestine by the leukemic cells in 13–25% of autopsy specimens [3]. The relatively common surgical complications noted in the intestine due to leukemia include typhlitis (necrotizing enterocolitis), appendicitis, haemorrhage and intestinal perforation. Intussuscpetion in ALL remains a very rare and an even poorly documented occurrence [4]. Rarely, cases of acute myeloid leukemia in adults have also presented with intussusception [5]. It is usually seen in patients who have started chemotherapy, rather than as complication of the disease process itself [6]. Here we present a newly diagnosed case of ALL in a child who developed intussusception prior to starting chemotherapy.

## Case presentation

A 14 year old male presented to the pediatric out-patient department with complaints of high grade fever, on and off, since 3 months along with swelling on both side of the neck since 2 months. The patient also complained of severe pain abdomen over the right iliac fossa, vomiting and an episiode of bleeding per rectum on the previous day. An episode of epistaxis was also noted. There was a history of significant weight loss and anorexia. On physical examination, pallor and pedal edema were noted. Multiple discrete subcentimetric lymph nodes were noted in bilateral cervical and inguinal regions. Multiple purpuric rashes were seen all over the body. Abdomen was tender with absent bowel sounds. An ill-defined lump was palpated over the right lower quadrant. Liver and spleen were palpated, 7 cm and 2 cm below right and left costal margins, respectively.

Complete hemogram revealed pancytopenia (Hb: 7.3 g/dl,TLC: 1500/cu. mm, DLC: Polymorphs 18%, Lymphocytes 72%, Eosinophils 1%, Monocytes 3%, Blasts 6%, Platelets 10,000/ cu.mm). Peripheral smear examination showed a predominantly normocytic normochromic blood picture with leucopenia and presence of a few circulating blasts. These blasts had high N: C ratio, indented nuclear margins, fine granular chromatin, with inconspicuous to occasional conspicuous nucleoli and minimal cytoplasm. The patient was admitted with a provisional diagnosis of pancytopenia with hepatosplenomegaly and lymphadenopathy under evaluation. To attain hemodynamic stability, eight units of Packed RBCs and four units of platelet rich plasma were transfused. Fine needle aspiration cytology done from the cervical lymph node showed reactive lymphadenitis. Ultrasonography of the abdomen was done to rule out probable causes of severe pain abdomen and lump in the right lower quadrant. This revealed an ileo-colic intussusception along with hepato-splenomegaly.

**Fig. 1:**
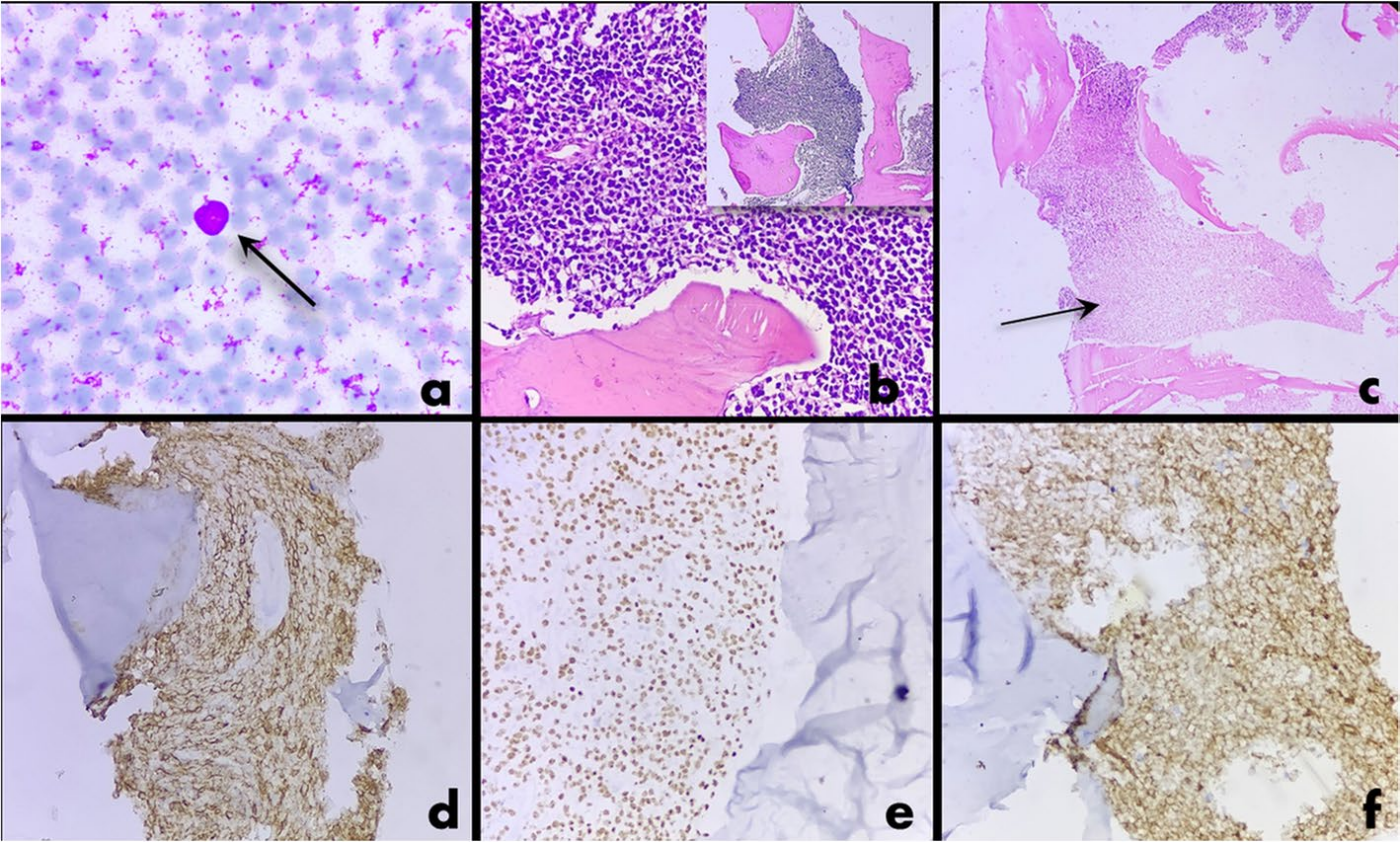
**a** An occasional blast in peripheral smear (arrow) (Leishmann stain, 1000x). **b** Photomicrograph of bone marrow biopsy showing cellular marrow spaces replaced by sheets of blasts (H&E, 400x). Inset: Low power view of the same area (H&E, 100x). **c** Photomicrograph of bone marrow biopsy showing extensive necrosis (arrow) (H&E, 100x). **d** Diffuse membranous staining of blast cells by CD 20 (CD 20 IHC, 400x). **e** Diffuse nuclear staining of blast cells by TdT (TdT IHC, 400x). **f** Diffuse membranous staining of blast cells by CD10 (CD 10 IHC, 400x)

In view of pancytopenia, bone marrow examination was done. Bone marrow aspirate yielded a dry tap. Smears from marrow imprint were markedly paucicelluar with marked degenerative changes, rendering a definitive comment impossible. Bone marrow biopsy showed hypercellular marrow spaces with complete effacement of normal architecture by sheets of blasts with similar morphology as in peripheral smear. Large areas of bone marrow necrosis were also noted (Grade II). Erythroid, megakaryocytic and granulocytic series of cells were markedly suppressed. Immunohistochemistry (IHC) of these immature cells revealed diffuse positivity for TdT, CD10 and CD20 while CD34 and CD117 were negative. Bone marrow biopsy and IHC findings were consistent with Common ALL antigen (CALLA) positive B-ALL. (Fig. 1).

The biochemical parameters including serum bilirubin, aspartate transaminase (AST)/ alanine transaminase (ALT), blood urea, serum creatinine and total proteins were within normal limits. Serum alkaline phosphatase was raised (428 IU/L).

After the diagnosis of B-ALL on bone marrow examination, the patient was started on i.v. Dexamethasone and i.v. Amikacin. Supportive treatment in the form of blood transfusion and I.V fluids were administered. Repeat CBC showed worsening of anemia (Hb: 5.4 gm/dl), leucopenia and thrombocytopenia. Surgery for intussusception was planned after stabilizing the patient. However, the patient succumbed to his condition two days after initiation of steroid therapy.

## Discussion

Intussusception is the telescoping of a proximal segment of the gastrointestinal tract within the lumen of the adjacent segment. Abdominal pain is the most common presenting symptom, followed by vomiting and bleeding from the rectum. Patients may also present with an abdominal mass or intestinal obstruction [7]. The occurrence of intussusception in Acute Leukemias has rarely been reported. The present case of ALL showed all of the classical signs and symptoms of intussusception.

A wide spectrum of gastrointestinal lesions has been documented in patients with acute leukemias. Prolla et al. studied 148 cases of acute leukemias and divided these lesions into 4 categories; haemorrhagic, leukemic, agranulocytic and fungal. The most common “leukemic” complications include plaque-like thickenings, nodular lesions, and polypoidal masses [3]. These locally raised mucosal lesions rarely cause an intussusception on their own. The pathogenesis is actually due to the lymphoid tissue which acts as a lead point in the initiation process for the formation of an intussusception [8]. Chien et al. studied 364 patients, who were diagnosed with acute leukemia and found that only 11 patients (3%) had developed acute abdominal complications, including intussusception. Among the other noted complications were acute appendicitis, intestinal perforation and ovarian cyst rupture [9]. Other studies have listed typhlitis and bowel inflammation due to chemotherapy as other uncommon causes of acute abdomen in patients of ALL [10]. It is however noteworthy to mention that the pathogenesis of intussusception in ALL may also be due to benign, non-leukemic lymphoid collections of considerable size which acts as a lead point [11]. In the present case, surgery could not be performed due to the unstable condition of the patient. However, based on the previous studies we can safely postulate that the lead point for intussusception could have been either an elevated mucosal leukemic infiltrate, an adjacent benign polypoidal lymphoid aggregate or a hematoma secondary to the leukemic process.

Shalaby et al. studied 9 cases of intussusception in ALL in children from 1930 to 2005. The study revealed that ileo-colic intussusception was the most common site as compared to colo-colic or ceco-colic. The lead point in ileocolic lesions is found in only 5% of the cases, which can lead to delay in diagnosis. Most of these cases were seen at the beginning of the induction therapy. As non-surgical methods for treatment of intussusception (enema) is usually not helpful nor advised for leukemic children, surgery is the most appropriate line of treatment [12]. However, in the present case, patient expired before surgery could be attempted.

Bone marrow necrosis (BMN) in acute leukemias is infrequent. It usually denotes a poorer prognosis. A study by Badar et al. included a total of 640 patients with ALL and 1,051 patients with AML, where the incidence of BMN was 3.2% and 2.4%. The study showed that the rate of Complete Remission to be inferior in patients with BMN (72%), as compared to patients without BMN (90%) [13]. In the present biopsy, Grade II BMN (20%-50% of total marrow space) was noted.

One of the possible documented causes of intussusception in patients of ALL is therapy induced [14]. Vincristine is usually a part of the induction therapy in ALL patients. It can predispose to therapy mediated ileus progressing to an adynamic length of the bowel. As the stool passage continues, the static portion of the bowel becomes edematous and septic. This can lead to an intussusception in itself [6]. In our case patient presented with intussusception prior to diagnosis of ALL and starting of treatment thereby ruling out this pathogenesis. Till date, no studies have been done to differentiate the relative risks of gastrointestinal complications between B-ALL and T-ALL. A comparison of various studies documenting intussusception in children with ALL has been tabulated. (Table 1).

**Table 1:** Comparison of different reported cases of intussusception in children with Acute Lymphoblastic Leukemia

Serial	Name of study (year)	Number of cases	Location	Cause	Treatment Stage	Treatment and Outcome
1	Shalaby et al. (2014) [12]	1	Left sided Colo-colic	No lead point seen	Three days prior to completion of induction	Surgical reduction. No recurrence
2	Shah et al. (2006) [15]	2	Case 1: Ceco-colic Case 2: Ileo-colic	Possible correlation between high dose methotrexate and intussusception	Case 1: 9^th^ week of consolidation Case 2: 14^th^ week of consolidation	Case 1 and Case 2: Reduction by barium enema followed by supportive treatment
3	Arestis et al. (2005) [6]	2	Case 1: Ileocecal Case 2: Descending colon	Case1: Vincristine mediated ileus Case 2: Non leukemic lymph node acting as lead point	Case 1: Day 8 of induction Case 2: Day 10 after completion of 13 cycles of vincristine	Case 1: Surgical reduction. No recurrences Case 2: Laparotomy with Hemicolectomy
4	Manglani et al. (1998) [2]	1	Ileo-cecal	Necrotic mucosa actin as lead point after leukemic infiltration	Day 4 of induction	Surgery followed by chemotherapy and bone marrow transplant. Patient could not be saved
5	Gavan et al. (1994) [11]	2	Case 1: Ileo-colic Case 2: Ceco-colic	Case 1: Enlarged lymph nodes as lead point Case 2: Unknown	Case 1: Day 11 of induction Case 2: Day 8 of induction	Case 1: Irreducible lesion. Treatment not documented Case 2: Surgical reduction
6	Micallef-Eynaud (1990) [4]	1	Ceco-colic	Vincristine associated bowel dysfunction along with typhlitis	Day 8 of induction	Surgical reduction followed by antibiotics for sepsis. Patient attained remission
7	Feldman et al. (1963) [16]	1	Upper Ileum	Leukemic infiltration of mucosa causing a hematoma	9 months after initiation of mercaptopurine	Antibiotics and supportive therapy. Patient succumbed in 48 h

Preoperative diagnosis of intussusception is challenging. Ultrasonography is a reliable screening tool and shows characteristic “pseudo-kidney” or “hay-fork” signs in the longitudinal view and “doughnut” or “target” signs in the transverse view [17]. However, enema is the gold standard for diagnosing intussusception in children which is both diagnostic and also therapeutic in many cases. In our case, as the child was severely immunocompromised enema couldn’t be performed. Computed tomography (CT) remains the modality of choice for assessing acute abdomen in adults. Based on plane of imaging, the “bowel within bowel” configuration when imaged at right angle to lumen and “soft tissue sausage like configuration” when viewed longitudinally is the classical CT finding in intussusception [18]. Although intussusception is rare in patients with ALL, a high index of suspicion and early diagnosis is of utmost importance as it has a significant effect on the patient’s prognosis.

## Conclusion

Intussusception is a recognized but a very rare complication of ALL. It should be suspected in all cases of ALL who presents with characteristic diagnostic symptoms. Conversely, in children with abdominal symptoms, weakness and deranged blood counts, the possibility of acute leukemia should always be considered. Delay in diagnosis usually leads to a poorer outcome. Usually older children with ALL have reported surgical complications. Both the clinician and the hematopathologist need to be aware of this potentially lethal but treatable condition for optimum and effective patient care.

## Data Availability

The data for current study is available from corresponding author at reasonable request.

## Abbreviations

ALL: Acute Lymphoblastic Leukemia
IHC: Immunohistochemistry
CALLA: Common ALL Antigen
CT: Computed Tomography
